# Supplementary material for: High-resolution crossover mapping reveals similarities and differences of male and female recombination in maize
Source: Nat Commun. 2018 Jun 18;9:2370. doi: 10.1038/s41467-018-04562-5 (PMC6006299; doi:10.1038/s41467-018-04562-5)
Supplement: Supplementary file 1 — Supplementary Information [file 41467_2018_4562_MOESM1_ESM.docx]

High-resolution crossover mapping reveals similarities and differences of male and female recombination in maize

Kianian *et al*.

**Male**

**Female**

**1**

**Genome coverage (x)**

**5**

**4**

**3**

**2**

**0**

Individuals

**Supplementary Figure 1. Genome coverage by Illumina-sequencing of plants genotyped for CO mapping.** Each bar represents a BC_1_ plant.

**Chromosome 1**

**Chromosome 2**

**Chromosome 3**

**Chromosome 4**

**Chromosome 5**

**Chromosome 6**

**Chromosome 7**

**Chromosome 8**

**Chromosome 9**

**Chromosome 10**

**Supplementary Figure 2. Distribution of SNPs between B73 and Mo17.**

**Chromosome 1**

**Chromosome length**

**Chromosome 6**

**Chromosome 3**

**Chromosome 2**

**Chromosome 4**

0

10

0

50

100

150

200 Mbp

5

0

20

10

30

0

50

100

150

200 Mbp

0

10

15

0

50

100

150

200 Mbp

5

0

10

15

0

50

100

150

200 Mbp

5

0

10

0

50

100

150 Mbp

5

0

10

15

0

50

100

150 Mbp

5

0

10

20

0

50

100 Mbp

0

20

10

30

50

100

150

200

250

300 Mbp

0

0

50

100

150 Mbp

5

0

80

120

40

0

160

Distance (cM)

80

40

0

Distance (cM)

80

40

0

120

Distance (cM)

80

40

0

Distance (cM)

80

40

0

Distance (cM)

80

40

0

Distance (cM)

80

40

0

Distance (cM)

80

40

0

Distance (cM)

80

40

0

Distance (cM)

CO rate

(cM/Mbp)

CO rate

(cM/Mbp)

CO rate

(cM/Mbp)

CO rate

(cM/Mbp)

CO rate

(cM/Mbp)

CO rate

(cM/Mbp)

CO rate

(cM/Mbp)

CO rate

(cM/Mbp)

CO rate

(cM/Mbp)

**Chromosome 5**

**Chromosome 8**

**Chromosome 9**

**Chromosome 10**

**Supplementary Figure 3. CO landscape on maize chromosomes 1 – 6 and 8 - 10.** See Figure 1 for chromosome 7. Blue = male COs, red = female COs. Colors on the X-axis indicate chromosome regions: purple = functional centromeres (= CENH3-binding regions), black = centromere repeats, gray = pericentromeric regions, light gray = distal regions, red = NOR and knobs. Insets are cumulative genetic distances across entire chromosomes. Gray triangles in insets are supplemental SNPs added, where needed, to create 1 Mbp intervals. Figures were generated using MareyMap.

**Supplementary Figure 4. Chromatin features of genes located at CO sites.** Random genes is a set of randomly selected 1000 genes.

**Supplementary Figure 5. Comparison of features of chromatin from isolated meiocytes/anthers and seedlings at sites of COs in male and female meioses.**

**Supplementary Figure 6. Relationship of CO sites in the B73 x CML228 hybrid relative to H3K4me3 patterns in the B73 and CML228 inbreds.**

Plant 1

HMM fit


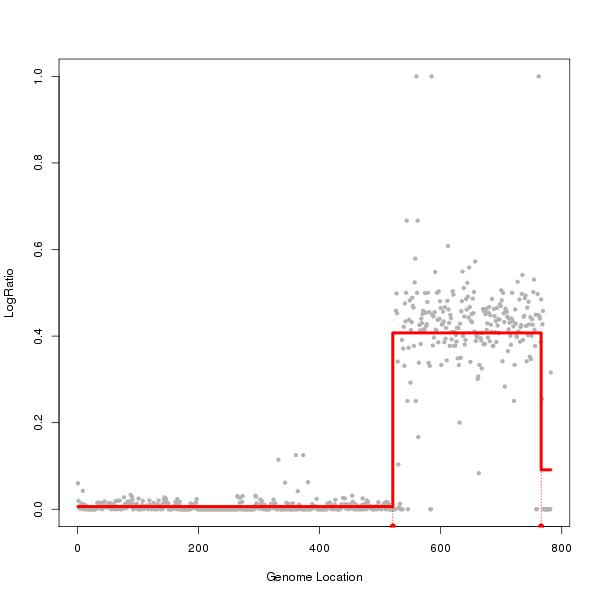


10 Mb

*PP* = 0 (Heterozygous)

*PP* = 1 (B73 homozygous)

Plant 1

Genome scaffold

Plant 2

Plant 3

=

**SNPs**

B73

homozygous

Heterozygous

Plant 2

Plant 3

Posterior probability

**A**

**B**

**C**

**D**

**Supplementary Figure 7. Bioinformatic pipeline used to identify CO positions.** (**A**) The mean-shift method was used to identify bins with similar Mo17 allele frequencies, and segment breakpoints were placed where neighboring bins showed significant allelic frequency differences. (**B**) An example of a 10-Mb segment breakpoint region showing Illumina reads aligned to the maize reference genome. Different colors indicate reads matching different inbred parents. (**C**) HMM was used to determine the origin of chromosomal segments (red = homozygous for B73, red/blue = B73/Mo17 heterozygous). (**D**) Posterior probability (PP) that a region is homozygous for B73 plotted on the y-axis with values ranging from 0 to 1. Region within the yellow dashed-line box represent PP confidence bounds for CO position.

**Supplementary Table 1. Comparison of genetic length of chromosomes in male and female meioses.**

| Chromosome | Male (cM) | Female (cM) | Male/female ratio |
| --- | --- | --- | --- |
| 1 | 154.9 | 174.9 | 0.89 |
| 2 | 92.5 | 93.6 | 0.99 |
| 3 | 116.4 | 113.2 | 1.03 |
| 4 | 88.9 | 104.1 | 0.85 |
| 5 | 71.9 | 85.0 | 0.85 |
| 6 | 73.5 | 75.6 | 0.97 |
| 7 | 81.9 | 92.6 | 0.88 |
| 8 | 83.1 | 93.7 | 0.89 |
| 9 | 95.2 | 94.5 | 1.01 |
| 10 | 82.7 | 77.0 | 1.07 |
| Total | 941.0 | 1004.2 | 0.94 |

**Supplementary Table 2. Size of chromosome end regions lacking COs.**

| Chromosome | Left end (Mbp) | Right end (Mbp) |
| --- | --- | --- |
| 1 | 2.2 | 1.9 |
| 2 | 2.5 | 3.1 |
| 3 | 2.2 | 2.2 |
| 4 | 2.2 | 4.7 |
| 5 | 3.4 | 14.8 |
| 6 | 1.7 | 1.5 |
| 7 | 3.1 | 3.0 |
| 8 | 0.6 | 3.0 |
| 9 | 2.4 | 3.3 |
| 10 | 0.5 | 1.5 |

**Supplementary Table 3. Statistically significant GO term enrichment in genes located within 10 kbp from male and female CO sites.**

| GO accession | Term | % of genes in sample | *P* value | FDR |
| --- | --- | --- | --- | --- |
| MALE | | | | |
| GO:0065007 | biological regulation | 24.8 | 0.00043 | 0.038 |
| GO:0043412 | macromolecule modification | 14.5 | 0.00028 | 0.024 |
| GO:0006796 | phosphate metabolic process | 14.1 | 0.00021 | 0.018 |
| GO:0006793 | phosphorus metabolic process | 14.1 | 0.00021 | 0.019 |
| GO:0006464 | protein modification process | 14.1 | 0.0003 | 0.026 |
| GO:0016301 | kinase activity | 13.8 | 0.0005 | 0.037 |
| GO:0016310 | phosphorylation | 13.7 | 9.80E-05 | 0.009 |
| GO:0016773 | phosphotransferase activity | 13.7 | 0.0002 | 0.018 |
| GO:0043687 | post-translational protein modification | 13.4 | 0.0004 | 0.039 |
| GO:0004674 | protein serine/threonine kinase activity | 12.6 | 2.60E-05 | 0.002 |
| GO:0006468 | protein amino acid phosphorylation | 12.6 | 7.80E-05 | 0.007 |
| GO:0004672 | protein kinase activity | 12.6 | 0.0001 | 0.008 |
| GO:0045454 | cell redox homeostasis | 3.1 | 0.0004 | 0.031 |
| GO:0015035 | protein disulfide oxidoreductase activity | 2.3 | 0.0002 | 0.017 |
| GO:0015036 | disulfide oxidoreductase activity | 2.3 | 0.0003 | 0.020 |
| FEMALE | | | | |
| GO:0016491 | oxidoreductase activity | 14.9 | 0.00032 | 0.025 |
| GO:0048037 | cofactor binding | 6.5 | 0.00059 | 0.046 |

*P* values were calculated using the Fisher's exact test. FDR = False discovery rate.

**Supplementary Table 4. Distribution of all COs and COs mapped to within 2 kbp or less in different chromosome regions.**

| Regions | All COs | Subset COs |
| --- | --- | --- |
| Distal | 71% | 74% |
| Pericentromeric | 27% | 25% |
| Centromeric | 1.80% | 1.40% |
