# Supplementary material for: High-resolution crossover mapping reveals similarities and differences of male and female recombination in maize
Source: Nat Commun. 2018 Jun 18;9:2370. doi: 10.1038/s41467-018-04562-5 (PMC6006299; doi:10.1038/s41467-018-04562-5)
Supplement: Supplementary file 3 — Description of Additional Supplementary Files [file 41467_2018_4562_MOESM3_ESM.pdf]

Descriptions of Additional Supplementary Files

File Name: Supplementary Data 1
Description: This file contains the list of SNP associated with COs in male and female meioses in the B73 x Mo17 hybrid.

File Name: Supplementary Data 2
Description: This file contains the list of recombination hotspots (i.e. regions 5 kbp in length exhibiting CO rates at least five-fold higher than the genome average1) in male and female meioses in the B73 x Mo17 hybrid.
